# Relationship between serum anion gap and mortality in ICU in multiple myeloma patients in the MIMIC database: A retrospective cohort study

**DOI:** 10.1371/journal.pone.0328014

**Published:** 2025-07-10

**Authors:** Qianhui Wang, Pengyu Hu, Haibo Cong

**Affiliations:** 1 The First Clinical Medical College of Shandong University of Traditional Chinese Medicine, Jinan, China; 2 Limin Hospital of Weihai High District, Weihai, China; Sutter Gould Medical Foundation, UNITED STATES OF AMERICA

## Abstract

**Background:**

Serum anion gap has diagnostic value in patients with multiple myeloma, but its association with ICU mortality and threshold value remain unclear.

**Methods:**

Multiple myeloma patients meeting criteria were selected from the Medical Information Mart for Intensive Care IV (MIMIC-IV) database. The exposure factor was serum anion gap, and the outcome was ICU in-hospital mortality. Multivariable-adjusted Cox regression, curve fitting, and forest plots were used to evaluate the relationship between anion gap and ICU mortality in multiple myeloma patients.

**Results:**

A total of 323 eligible subjects were included (206 males [63.8%], 117 females [36.2%]). Multivariable Cox regression showed that each 1-unit increase in AG was associated with a 7% increased mortality risk (HR = 1.07, 95%CI = 1.01–1.14, P = 0.032). Curve fitting revealed a nonlinear relationship between anion gap and ICU mortality (nonlinear P = 0.038), with the lowest risk at 15.29 mmol/L. Incorporating AG into traditional risk factor models improved mortality prediction (P = 0.038).

**Conclusion:**

Serum anion gap exhibits a nonlinear relationship with ICU mortality in multiple myeloma patients, with the lowest risk observed at approximately 15.29 mmol/L.

## Introduction

Serum anion gap (AG) is an important blood biomarker that provides valuable guiding principles for human physiological status and overall health. AG is a commonly used clinical or statistical calculation index, which approximates the concentration difference between unmeasured anions (UA) and unmeasured cations (UC) in serum [1]. Chronic conditions such as hypertension, diabetes, and heart disease are risk factors for developing complications (e.g., lactic acidosis, ketoacidosis) that elevate serum anion gap due to unmeasured anions [2,3]. AG is also a simple, economical, and effective monitoring indicator for detecting acid-base imbalances in bodily functions, evaluating laboratory parameters to facilitate clinical monitoring, and tracking hematologic malignancies (multiple myeloma, MM) and various intoxication issues [4]. Studies by foreign scholars have shown that the SOFA score has only moderate predictive value for mortality within 7 days after admission, but the SOFA-AG score demonstrates higher predictive capability for mortality within 90 days after admission [5], indicating that combining the SOFA score with the AG significantly enhances predictive capacity.

Multiple myeloma (MM) is a biologically heterogeneous plasma cell disorder [6], an incurable hematologic malignancy characterized by the accumulation of abnormal plasma cells in the bone marrow [7]. Diao Xiang wen et al. [8] reported that the prognosis of MM patients admitted to the ICU is primarily determined by the severity of organ failure, infections, and disease status. Furthermore, studies have demonstrated that the AG is associated with mortality in cardiac intensive care patients [5]. Identifying effective predictors of in-hospital mortality for critically ill MM patients holds significant clinical importance, enabling physicians to better diagnose the disease, communicate promptly with patients and their families, and facilitate rational allocation of medical resources.

Research experiments by scholars have shown that compared to the control group (non-MM patients), MM patients exhibit significantly reduced AG levels [9]. However, the effectiveness of AG in monitoring MM patients requires further research validation [9]. Therefore, the purpose of this study is to investigate the potential correlation between the exposure factor AG and ICU in-hospital mortality, and to determine the threshold level at which AG may influence ICU in-hospital mortality. This retrospective cohort study included 323 MM patients from the MIMIC database.

## Materials and methods

### MIMIC-IV database

This is a retrospective study derived from the MIMIC-IV database (version: 2.2) [10], which contains detailed data from over 40,000 patients admitted to the intensive care unit at Beth Israel Deaconess Medical Center in Boston, Massachusetts, between 2008 and 2019. Anyone who passes the Collaborative Institutional Training Initiative exam and applies for database access can use the database (certification number for Qian hui Wang: 52968321). As this is a retrospective cohort study, the patients included in this study were all extracted from a public database, ensuring certain privacy and confidentiality, thus waiving the requirement for informed consent [11].

The establishment of this database was approved by the Massachusetts Institute of Technology (Cambridge, MA) and Beth Israel Deaconess Medical Center (Boston, MA), and consent was obtained for the original data collection. Therefore, the ethical approval statement and the need for informed consent were waived for this manuscript.

### Population

**Fig 1:**
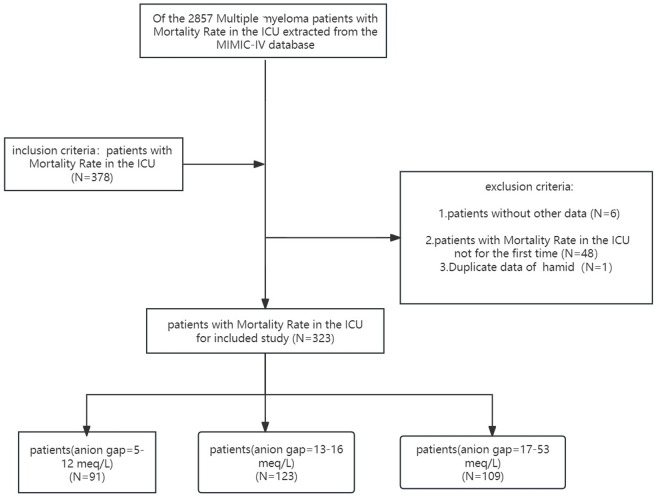
Flowchart of study population.

MM patients from 2008 to 2019 were selected from the MIMIC-IV database. Inclusion criteria were as follows: adult MM patients admitted to the ICU; MM was defined using ICD-9 codes 20300, 20301, 20302 or ICD-10 codes C9000, C9001, C9002. Among the 2,857 patients initially extracted from the MIMIC-IV database, 2,479 were excluded due to lack of ICU admission records. Additionally, 48 patients were excluded for multiple ICU admissions, and we retained only the first ICU admission for MM patients, excluding one duplicate record. To mitigate the impact of missing values, six patients with missing covariates were excluded. Ultimately, 323 MM patients were included in this study (Fig 1). All valid patient information was extracted from the corresponding tables in the MIMIC-IV database [12].

### Covariates

AG included was the first AG measurement within 24 hours of ICU admission for MM patients in the MIMIC-IV database. AG level in adults is approximately 3–11 mEq/L [1]^(1)^. The exposure factor in this study was AG, and the outcome factor was in-hospital mortality following ICU admission. Variables incorporated into the database included gender, age, race, highest heart rate, highest systolic blood pressure, highest diastolic blood pressure, highest respiratory rate, highest body temperature, lowest oxygen saturation, lowest hematocrit, lowest hemoglobin, lowest platelet count, highest white blood cell count, highest AG, highest blood glucose, creatinine, Simplified Acute Physiology Score II (SAPS II is a validated ICU mortality prediction model comprising 17 variables (12 physiological, 3 chronic disease, 2 admission-type parameters [13–15].), and Oxford Acute Severity of Illness Score (OASIS is a parsimonious ICU risk stratification tool using 10 electronically retrievable parameters. Specifically calibrated for MIMIC database populations.). Incorporating both SAPS II and OASIS provides complementary risk assessment.

### Statistical methods

All participants underwent descriptive analysis. Continuous variables were described as mean ± standard deviation (SD) or median and interquartile range (IQR), while categorical data were described as frequencies or percentages. The Mann-Whitney U test was used for continuous variables, and the chi-square test was used for categorical variables. Methods such as curve fitting, multivariable Cox regression analysis, threshold effect analysis, and forest plots were employed to evaluate the independent correlation and threshold between AG and ICU mortality in MM patients. The Cox model approach was extended to models adjusted for various covariates. Covariates were selected based on prior research findings and clinical constraints. Alternatively, covariate analysis was used, with variables selected for adjustment if the matched odds ratio changed by at least 10%.

Based on AG levels in the study, patients were divided into three tertiles: 5–12 mEq/L, 13–16 mEq/L, and 17–53 mEq/L. Model I was adjusted for age and race. Model II was adjusted for age, race, highest heart rate, highest respiratory rate, lowest platelet count, highest white blood cell count, highest blood glucose, SAPS II, and OASIS. Results were expressed as hazard ratios (HR) with 95% confidence intervals (CI).

All statistical analyses were performed using R Statistical Software (version 4.2.2, http://www.R-project.org, R Foundation) and the Free Statistical Analysis Platform (version 1.9, Beijing, China, http://www.clinicalscientists.cn/freestatistics) [16].

## Results

### Baseline characteristics of study participants

The study included 323 eligible participants from the MIMIC-IV database (Fig 1). Previous experimental results confirmed a 32% mortality rate for critically ill cancer patients in the ICU [17]. Table 1 displays the general characteristics of patients stratified by AG tertiles. The mean patient age was 72.1 ± 11.2 years, with 206 males (63.8%) and 117 females (36.2%). AG at the first ICU admission was 15.4 ± 5.2 mEq/L. AG were positively correlated with race, creatinine, SAPS II score, and OASIS score. The mean SAPS II score on admission day 1 was 48.6 ± 12.3, and the mean OASIS score was 32.3 ± 8.4. No statistically significant differences were observed in other general characteristics (all *P* > 0.05).

**Table 1:** Baseline demographic characteristics of the study population stratified by AG.

Variables	Total (n = 324)	Anion gap(mmol/L)			*p*
		5-12 (n = 91)	13-16 (n = 123)	17-53 (n = 110)	
Gender, n (%)					0.371
Female	117 (36.2)	32 (35.2)	40 (32.5)	45 (41.3)	
Male	206 (63.8)	59 (64.8)	83 (67.5)	64 (58.7)	
age, y	72.1 ± 11.2	71.4 ± 10.6	73.2 ± 11.2	71.5 ± 11.8	0.400
race, n (%)					**0.006**
White	208 (64.4)	71 (78)	74 (60.2)	63 (57.8)	
Other	115 (35.6)	20 (22)	49 (39.8)	46 (42.2)	
Highest HR (bmp)	107.4 ± 21.0	104.3 ± 19.4	106.3 ± 20.9	111.0 ± 22.1	0.062
Highest SBP (mmHg)	144.9 ± 22.0	143.4 ± 21.5	145.6 ± 22.2	145.3 ± 22.3	0.739
Highest DBP (mmHg)	88.7 ± 19.0	87.4 ± 18.8	88.8 ± 17.0	89.7 ± 21.2	0.685
Highest RR (bmp)	29.5 ± 6.9	29.9 ± 7.6	29.2 ± 6.6	29.6 ± 6.8	0.779
Highest temperature (°C)	37.4 ± 0.7	37.5 ± 0.8	37.3 ± 0.7	37.3 ± 0.7	0.069
Lowest SPO2 (%)	91.1 ± 8.6	90.5 ± 9.8	91.6 ± 8.3	90.9 ± 7.7	0.668
Lowest HCT (%)	26.5 ± 5.8	26.2 ± 5.2	26.7 ± 6.3	26.5 ± 5.9	0.878
Lowest HB (g/dL)	8.7 ± 1.9	8.7 ± 1.7	8.8 ± 2.0	8.7 ± 2.0	0.929
Lowest PLT(K/UL)	132.0 (73.5, 193.5)	123.0 (72.0, 205.0)	131.0 (66.5, 184.5)	146.0 (92.0, 194.0)	0.602
Highest WBC(K/UL)	8.0 (4.8, 12.0)	7.7 (4.6, 12.0)	8.1 (5.0, 12.0)	8.1 (4.9, 12.2)	0.911
Highest AG (mEq/L)	15.4 ± 5.2	10.5 ± 1.7	14.4 ± 1.2	20.7 ± 5.0	**< 0.001**
Highest glucose	140.0(116.0,171.0)	140.0 (121.5, 164.5)	132.0 (114.0, 166.5)	142.0 (112.0, 182.0)	0.497
Cre (mg/dl)	1.6 (1.0, 3.0)	1.2 (0.8, 1.6)	1.6 (1.0, 2.8)	2.8 (1.6, 5.6)	**< 0.001**
SAPSII,	48.6 ± 12.3	44.7 ± 11.4	47.5 ± 10.6	53.2 ± 13.5	**< 0.001**
Oasis,	32.3 ± 8.4	31.5 ± 8.4	31.2 ± 8.2	34.2 ± 8.4	**0.016**

Notes: Data presented are mean ± SD, median (IQR), or N (%).

Abbreviations: SD: standard deviation; IQR: interquartile range; HR: heart rate; SBP: systolic blood pressure; DBP: diastolic blood pressure; RR: Respiratory Rate; HCT: hematocrit; HB: hemoglobin; PLT: Platelet; WBC, white blood cell; AG: Anion gap; Cre: creatinine; SAPS II: Simplified Acute Physiology Score II; OASIS: Oxford acute severity of illness score.

### Association between serum anion gap and prognosis

Table 2 summarizes the risk factors associated with ICU mortality in MM patients using univariate Cox analysis, reported as HR and 95% CI. Age, gender, highest heart rate, highest respiratory rate, lowest oxygen saturation, AG, highest blood glucose, SAPS II score, and OASIS score were significantly associated with ICU mortality (*P* < 0.05). Conversely, other laboratory results such as minimum hematocrit, minimum platelet count, and maximum white blood cell count, as well as other factors, showed no significant association with ICU mortality (Table 2).

**Table 2:** Association of covariates and in-hospital morality in patients with MM.

Variable	HR (95%CI)	P-value
gender: Male vs Female	0.56 (0.33,0.97)	**0.038**
age (cont. var.)	1.03 (1.01,1.06)	**0.02**
Race: Other vs White	1.31 (0.76,2.27)	0.333
Highest HR (bmp)	1.01 (1,1.03)	**0.032**
Highest SBP (mmHg)	0.99 (0.98,1)	0.091
Highest DBP (mmHg)	0.992 (0.9775,1.0067)	0.284
Highest RR (bmp)	1.04 (1,1.07)	**0.032**
Highest temperature (°C)	0.91 (0.63,1.32)	0.607
Lowest SPO2 (%)	0.98 (0.97,1)	**0.012**
Lowest HCT (%)	1.04 (0.99,1.09)	0.094
Lowest HB (g/dL)	1.09 (0.95,1.26)	0.217
Lowest PLT(K/UL)	0.9985 (0.9953,1.0017)	0.362
Highest WBC(K/UL)	1.003 (0.9708,1.0361)	0.859
Highest AG (mEq/L)	1.09 (1.04,1.14)	**< 0.001**
Highest glucose	1.0026 (1.001,1.0043)	**0.001**
Cre (mg/dl)	1.05 (0.96,1.15)	0.309
SAPSII	1.03 (1.01,1.04)	**< 0.001**
Oasis	1.03 (1.01,1.06)	**0.017**

**Fig 2:**
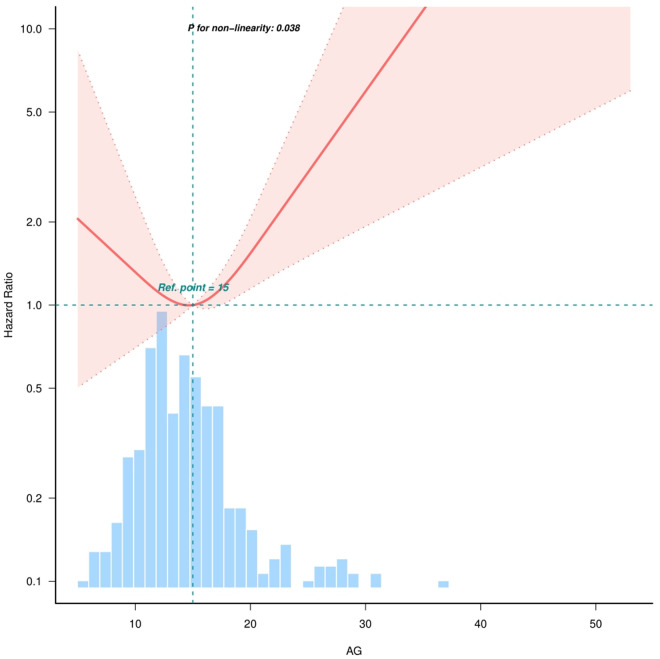
The nonlinear relationship between AG and in-hospital mortality in Multiple Myeloma in ICU. Adjusted for all covariates as model 2. (knots adjusted to 3).

Table 3 presents the results of the multivariable Cox proportional hazards model, showing the correlation between AG (as a continuous or categorical variable) and ICU mortality. When AG was treated as a continuous variable, each 1-unit increase in AG was associated with a 7% increased risk of ICU mortality (HR = 1.07, 95% CI = 1.01–1.14, *P* = 0.032), even after adjusting for potential confounders derived from clinical experience and covariate screening (Table 3, Fig 2). The association remained statistically significant after controlling for potential confounders (Table 3, Figs 1 and 2), though slightly attenuated (HR = 1.07, 95% CI = 1.01–1.14, *P* = 0.032).

**Table 3:** Relationship between different Anion gap levels and in-hospital morality in different models.

Anion gap	Non-adjusted Model		Model 1		Model 2	
	HR (95% CI)	P-value	HR (95% CI)	P-value	HR (95% CI)	P-value
Anion gap(mmol/L)	1.09(1.04 ~ 1.14)	<0.001	1.09 (1.04 ~ 1.14)	0.001	1.1 (1.03 ~ 1.17)	0.003
5-12 mmol/L	Reference		Reference		Reference	
13-16 mmol/L	1.11(0.54 ~ 2.29)	0.782	1.06 (0.51 ~ 2.2)	0.875	0.8 (0.38 ~ 1.69)	0.554
17-53 mmol/L	1.83(0.92 ~ 3.65)	0.087	1.68 (0.84 ~ 3.35)	0.143	1.42(0.65 ~ 3.08)	0.379

Notes: Data presented are HRs and 95% CIs.

Model I: adjusted for gender, age, race. Model II: adjusted for gender, age, race, HR, spo2, PLT, WBC, Cre, SAPSII.

### Nonlinear relationship and sensitivity analysis

To investigate potential nonlinearity between AG and ICU mortality, curve fitting and threshold effect analyses were performed. After adjusting for confounders, a nonlinear association was identified (nonlinear *P* = 0.038, Fig 2). ICU mortality risk was associated with AG levels, with the lowest risk at a threshold of 15.29 mmol/L (95% CI = 14.587–15.994), and a likelihood ratio test *P*-value < 0.046 (Table 4). When anion gap exceeded 15.29 mmol/L, the odds ratio for ICU mortality risk became 1.143 (95% CI = 1.032–1.265, *P* = 0.0101), revealing a nonlinear relationship between anion gap levels and ICU mortality risk. As shown in Fig 2, both low and high anion gap levels were associated with elevated ICU mortality risk.

**Table 4:** Threshold effect analysis of serum anion gap level and the in-hospital mortality of MM using Cox regression models.

	HR (95% CI)	P-value
Turning point(mmol/L)	15.29 (14.587,15.994)	
Anion gap < 15.29 mmol/L	0.865 (0.716,1.045)	0.1328
Anion gap>=15.29 mmol/L	1.143 (1.032,1.265)	0.0101
Likelihood Ratio test	–	0.046

### Stratified analysis based on covariates

**Fig 3:**
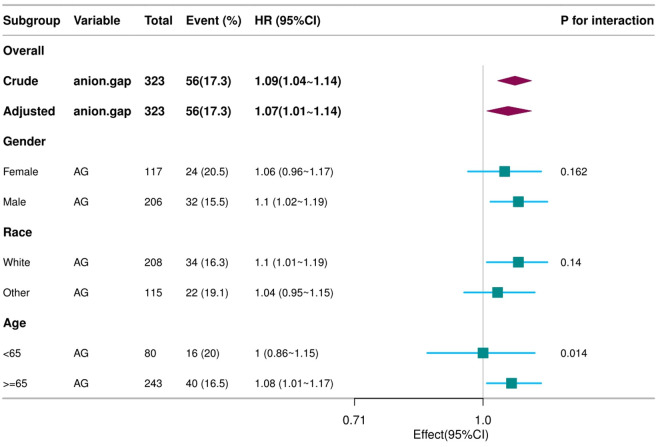
Forest plot shows HRs of in-hospital mortality in AG group using a variety of models. Except for the stratification component itself, each stratification factor was adjusted for all other variables (age, race, HR, RR, PLT, WBC, glucose, SAPSII, Oasis).

Stratified analyses across subgroups (e.g., gender, race, age) were conducted to assess potential influences on the AG–ICU mortality relationship. No significant interactions were observed among these subgroups (Fig 3).

## Discussion

In this retrospective cohort study of MM patients in the MIMIC database, we identified for the first time an independent association between AG and ICU mortality. Curve fitting analysis revealed a nonlinear relationship between AG levels and ICU mortality risk (nonlinear *P* = 0.038), with the lowest risk observed at 15.29 mEq/L. Importantly, incorporating AG into traditional risk factor models significantly improved the predictive capacity for ICU mortality. These findings have critical implications for managing MM patients in the ICU.

Epidemiological studies have confirmed that AG is an independent risk factor for ICU mortality. For example, a study based on the MIMIC-III database demonstrated that elevated AG serves as a surrogate parameter for predicting sepsis mortality when other urgent clinical indicators are unavailable, aligning with our findings [18]. Furthermore, pooled results from epidemiological studies report AG as an independent predictor of mortality across diverse conditions [19–23]. In our cohort, the nadir of the nonlinear relationship between AG and ICU mortality in MM patients corroborates that both high and low AG levels may contribute to increased mortality, warranting further investigation.

Beyond these established insights, our study highlights several notable points. First, the nonlinear relationship between AG and ICU mortality persisted even after adjusting for confounders. Second, the threshold value of 15.29 mEq/L, derived from threshold effect analysis, corresponds to the lowest mortality risk. Prior studies report that an initial AG > 16 mmol/L in critically ill patients is associated with elevated mortality [24], which aligns closely with our threshold value. In multiple myeloma (MM) patients, AG values below 15.29 mmol/L may reflect severe hypoalbuminemia, a common complication due to renal dysfunction or malnutrition [25]. Albumin (a major unmeasured anion) contributes ~75% of AG’s negative charge [26]. When albumin declines, AG decreases disproportionately [26], masking underlying acid-base disorders. Critically ill MM patients with low AG often exhibit advanced disease, systemic inflammation [27], and capillary leakage [28,29], exacerbating hypoalbuminemia. Aggressive fluid resuscitation in septic MM patients dilutes serum anions (e.g., albumin, lactate) [30,31], lowering AG. This dilution reflects hemodynamic instability and tissue hypoperfusion—key drivers of mortality [32,33]. Fluid overload >5% correlates with ICU mortality in hematologic malignancies [33,34]. These findings further support existing knowledge on MM patient outcomes and suggest that elevated AG may serve as a valuable predictor of ICU mortality in this population.

To date, only limited studies have explored the AG–mortality relationship in ICU settings. Our findings are consistent with prior work [35], as we similarly observed a nonlinear mortality trend in MM patients within the MIMIC database, even after adjusting for age, race, maximum heart rate, maximum respiratory rate, minimum platelet count, maximum white blood cell count, maximum blood glucose, SAPS II, and OASIS. Future studies are needed to validate these results and elucidate underlying mechanisms.

This study pioneers the elucidation of the correlation between AG levels and ICU mortality risk using MIMIC-IV data. Its strength lies in leveraging a rigorously validated database with comprehensive diagnostic, hospitalization, and ICU admission records, minimizing biases such as selection and recall bias common in observational studies.

However, several limitations must be acknowledged. First, the observational design and measurement methods preclude mechanistic insights. Second, the small sample size limits statistical power and exploration of potential interactions. The cohort’s restriction to U.S. residents necessitates caution when generalizing findings to other populations. Third, while our preliminary findings are insightful, validation in larger cohorts is essential to strengthen confidence in the conclusions. Fourth, residual confounding by unmeasured factors (e.g., diet, socioeconomic status) cannot be excluded. Fifth, while prior studies established that multiple myeloma (MM) patients exhibit lower baseline anion gap (AG) than non-MM populations due to paraprotein effects, this reduction is particularly characteristic of IgG-type MM. Our analysis reveals a J-shaped association between AG and ICU mortality [9]. To strengthen these conclusions, future studies should:

Stratify by immunotype to compare AG-mortality trends across IgG, IgA, and IgD myeloma cohorts; Incorporate etiology-specific variables, including: Measurement of lactate to isolate lactic acidosis contributions, application of KDIGO criteria to define acute kidney injury (AKI) stages, systematic tracking of glucose and ketones to identify diabetic ketoacidosis (DKA). Further research is required to confirm the utility of AG in monitoring MM patients. Sixth, we measured only the initial ICU admission AG, not dynamic changes, which merits future investigation.

Prospective multicenter studies are warranted to validate AG as a prognostic factor for adverse outcomes in critically ill MM patients. Further exploration of nonlinear trends between AG and MM, or interactions with other laboratory indices, could guide therapeutic strategies and predict intervention outcomes [36].

## Conclusion

Our data reveal a nonlinear relationship between AG and ICU mortality in MM patients, with the lowest risk observed at 15.29 mEq/L. These findings underscore the importance of AG as a potential risk factor for ICU mortality and highlight its utility as a biomarker. Further investigation is needed to deepen understanding of this association. The results require additional validation and confirmation.

## Abbreviations

ICU: intensive care unit
MM: Multiple myeloma
AG: Anion gap
SD: standard deviation
IQR: interquartile range
HR: heart rate
RR: Respiratory Rate
SBP: systolic blood pressure
DBP: diastolic blood pressure
HCT: hematocrit
HB: hemoglobin
PLT: Platelet
WBC: white blood cell
Cre: creatinine
SAPS II: Simplified Acute Physiology Score II
OASIS: Oxford acute severity of illness score
